# Adolescent Obesity Prevention in Saudi Arabia: Co-identifying Actionable Priorities for Interventions

**DOI:** 10.3389/fpubh.2022.863765

**Published:** 2022-05-10

**Authors:** Manal Almughamisi, Majella O'Keeffe, Seeromanie Harding

**Affiliations:** ^1^Department of Nutritional Sciences, King's College London, London, United Kingdom; ^2^School of Food and Nutritional Science, Biosciences Institute, University College Cork, Cork, Ireland; ^3^Department of Population Health Sciences and Department of Nutritional Sciences, School of Life Course and Population Sciences, Faculty of Life Sciences & Medicine, King's College London, London, United Kingdom

**Keywords:** adolescents, obesity, intervention, concept mapping, prevention

## Abstract

**Background:**

Childhood obesity is a serious issue in the Kingdom of Saudi Arabia, but there is no known community intervention. The aim of the study was to use a participatory approach to obtain the perspectives of students, school staff and Ministry of Education (MoE) representatives and parents on important and feasible intervention opportunities for school-based obesity prevention for adolescent girls.

**Method:**

The study was conducted in two intermediate schools for girls (13–15 years old) in Jeddah that were purposefully identified with the support of the MoE. Group concept mapping, a mixed method approach, was conducted with 19 adults which included staff from the MoE and schools, school canteen suppliers and mothers. Adults generated statements in response to two prompts (P); P1 “*The factors influencing adolescent obesity in Saudi are...”* and P2 “*The content of school-based programmes should focus on....”* Photovoice-enhanced concept mapping was used with students (*n* = 15 students) to capture adolescent perspectives on what influences their dietary and physical activity habits. Students generated statements' using their own photographs. Stakeholders, both adult and students, sorted the statements into themes and rated each statement for relative importance and feasibility. Multidimensional scaling and hierarchical cluster analyses were used to produce concept maps with the input from students and adults.

**Result:**

Adults generated 35 statements in response to P1 and identified five themes that influenced adolescent obesity including “Home Environment,” “Lifestyle,” “School Environment,” “Community,” “Biology.” They generated 42 statements in relation to P2 and identified four themes including “Ministry of Education Support,” “School Environment,” “Public health programmes” and “Wider environmental influences.” Students generated 42 statements from 39 pictures. They identified five themes that influenced their dietary and physical activity habits—“Role of Government,” “School Environment,” “Home Environment,” “Retail Environment” and “Cultural Practices.” Both groups identified several common important and feasible actions with a strong emphasis on improving the school environment, in particular food provision, with MoE support. Exemplar corresponding statements from adults were “*Offer healthy foods in the canteen,” “Remove chocolates and sweets”* and “*Educate children about healthy foods”* and from students were “*Offer fruit and vegetables in the canteen,” “Remove chocolates from the canteen,” “Healthy meals should not expensive.”* Lack of correspondence related to students' emphasis on access to both healthy foods and physical activity in schools and the wider environment (e.g. retail environments), while adults emphasized school-based education and food provision. After further consultations, both stakeholder groups agreed on improving access to healthy foods in the canteen.

**Conclusions:**

Students and school and MoE staff jointly agreed that a canteen-based intervention was important and feasible to improve dietary habits and thus help to prevent obesity among adolescent girls. This was the first time a participatory approach was used with students for intervention development in Saudi Arabia. A co-development approach may have value to improve their school food environments.

## Introduction

Childhood obesity is a major public health concern in the Kingdom of Saudi Arabia. The most recent data available in 2014 showed that ~1 in 3 boys and 1 in 4 girls aged 12–18 years old were either overweight or obese (1). The Saudi-based literature is scant but high intake of sugar-sweetened beverages, eating away from home, skipping breakfast and low hours of physical activity appear to be key drivers (2). Similar to patterns observed in low- and middle-income countries (3), there have been reports of a positive association between socioeconomic circumstances and overweight and obesity in Saudi (4, 5). In Saudi, all healthcare costs are government funded and recently The Saudi Vision 2030 recognizes the financial burden obesity is placing on government resources. As a result, regulatory interventions are emerging. As part of the 2030 Vision, an excise tax of 50% on carbonated beverages was implemented in 2017 and in 2018 energy information was required on menus in restaurants, including dine-in restaurants and fast-food restaurants (6). There are, however, no known community-level interventions, particularly those targeting young people. School-based interventions have the potential for effective obesity prevention and promotion (7). Most children spend half of their waking hours in school and consume a significant proportion of their daily calories at school.

Rapid economic development in Saudi has been driven by the oil boom of the 1970s (8). Saudi is one of the top twenty economies in the world and the largest in the Arab world and the Middle East (9). Household income increased by 75% between 2004 and 2013 and was largely driven by higher public sector employment and salaries (8). From a nutrition transition perspective (10), Saudi Arabia is considered to be in an advanced stage of transition where non-communicable diseases account for 78% of all deaths (11). Similar to low- and middle-income countries, there has been a shift away from diets composed of whole foods (e.g., pulses and whole grains) and low in refined oils and sugars to an energy-dense and nutrient-poor diet composed of fat and sugar-rich diets, and processed foods (12).

Jeddah, located in the western region of Saudi Arabia, on the coast of the Red sea is the second largest city in the Kingdom, with a population of 4,697,000 people after the capital Riyadh (13), and is an important commercial center. Saudi Arabia is an Islamic state and Jeddah is known as the gateway to the holy cities (Makkah and Medinah). As a result, Jeddah has historically attracted people from different parts of the Islamic world to settle there. Forty percent of the population of Jeddah are not native-born Arabic people, with migrants mainly from Southern Asia, particularly India, Pakistan and Bangladesh. In 2012, the prevalence of obesity in Jeddah was 35% in adults of both sexes, which was higher for Saudi Arabia overall in the 2013 national survey (14). In 2011, the prevalence of overweight and obesity combined among children and adolescents (ages 6–19 years) in Jeddah was 7.1% and 14.4% respectively. As mentioned, Saudi Arabia has made some effort with regards to obesity prevention such as the excise tax on carbonated beverages (15), energy declaration on the menus (6). However, there is a general lack of community health-promoting programmes, and more specifically co-designed programmes.

The value of co-production and participatory approaches is gaining rapid recognition in public health. This involves researchers, practitioners and the public working together from the outset to jointly develop knowledge that is actionable and that can catalyze transformation of systems (16, 17). Norström et al. defined co-production as “*Iterative and collaborative processes involving diverse types of expertise, knowledge and actors to produce context-specific knowledge and pathways towards a sustainable future”* (18). The issue of the participation of young people has also gained much recognition in policy, research, education and community development initiatives, and recently in driving forward the climate change agenda globally (19, 20). Young people can better identify the problem to be tackled because they understand the needs, experiences and capabilities of young people in ways that adults cannot. Integrating the perspectives of students with those responsible for the planning and provision of care, such as policy actors in education, parents and teachers, is thus key in the planning of school-based intervention programmes (21).

In this study we used concept mapping, which is steeped in theory-driven evaluation and participatory action research and is increasingly being used to identify and implement effective practices in public health (22, 23). It combines a structured qualitative and quantitative approach with stakeholders throughout the research process (24). It can be flexibly adapted to enhance participation of young people and generate a meaningful conceptualization of the issues being investigated (25). This paper reports on the perspectives of students, school staff, staff at the Ministry of Education, and a mother on the factors that influence adolescent obesity and on feasible priorities for the content of a school-based programme.

## Methods

### Setting and Sampling

This study was conducted in the city of Jeddah (KSA). Two intermediate schools for girls (13–15 years old) were purposefully identified with the support of the Ministry of Education. Intermediate schools capture Grades 1 (age 13 years) to 3 (age 15 years). In Saudi Arabia, schools are rated based on specific exams called “Qiyas.” These exams aim to detect students' potential abilities and academic skills in the fields of language, mathematics, science and creativity. Of the two schools included in the study one school was rated as below average and the other was rated as average/above average in terms of academic performance, based on Qiyas. Twenty-six students were randomly selected from each year of each school (13 from school 1- and 13 from school 2; 4 from Grade 1, 4 from Grade 2 and 5 from Grade 3 of each school) and invited to participate. Fifteen students agreed to participate (7 from school 1 and 8 from school 2); 90% with at least one parent employed, 44% with mothers who had a University degree. Eleven students did not take part and cited preparation for their examinations as the reason for non-participation. The socio-economic backgrounds of those who did not take part did not differ significantly from those who took part.

Twenty-five adults were purposely selected and invited to take part, 19 of whom agreed and included 10 teachers (five home economics and five science teachers), six administrative staff, two canteen staff, one mother of the students at the participating schools, and six Ministry of Education staff who were responsible for nutrition and health in the participating schools. Six participants (three teachers and three administrative staff) did not participate due to teaching commitments. Only one mother was able to join.

### Concept Mapping

Concept mapping enables shared understanding and provides “*balance of power”* as the method is predominantly participant-led rather than researcher-driven (26, 27). The method is described in detail elsewhere (24, 28). Concept mapping has six key steps: (1) Preparation, where stakeholders are identified, and the prompts are developed, (2) Generation, where stakeholders brainstorm a set of statements related to the prompt, (3) Structuring, where each participant sorts the statements into clusters based on perceived similarity of the statements, and also where each participant rates each statements for importance and for feasibility of action, (4) Representation, where the data are analyzed using multidimension scaling , (5) Interpretation, where derived maps are discussed with the group and (6) Utilization (not used in current study), where the concept maps can be used to plan interventions.

In the current study the following process was used for concept mapping. First, concept prompts were developed during initial informal consultations with teachers and students. Adults were then presented with these prompts and then generated statements in response to these; Prompt 1 “The factors influencing adolescent obesity in Saudi are...” and Prompt 2 “The content of school-based programmes should focus on....” Photovoice-enhanced concept mapping (29) was used in the student workshops to capture students' perspectives on what influences their dietary and physical activity habits. In brief, ten students were randomly chosen to take pictures using the “lucky dip” method. Folded slips of paper with the word “camera” were placed in a box and students were asked to pick a slip. Students with a “camera” slip were asked to take pictures of anything in their environments, home, school or elsewhere, that they felt influenced their dietary and physical activity habits. The pictures were then used as prompts to generate statements in a group session with all 15 students. Duplicated statements were removed, and some statements were amended to improve clarity on consultation with the students.

Each participant, both adults and children, then sorted the statements into clusters based on perceived conceptual similarity and provided a word/short phrase to describe the cluster. Following the sorting of the statements, each participant was given rating grids that contained all the generated statements. Participants were asked to rate each statement on a 5-point scale for importance and a 5-point scale for feasibility of achieving a positive change. The 5-point rating scales were: 1 = Relatively unimportant, 2 = Somewhat important, 3 = Moderately important, 4 = Very important, 5 = Extremely important, and for feasibility of achieving a positive change: 1 = Not at all feasible, 2 = Somewhat feasible, 3 = Moderately feasible, 4 = Very feasible, 5 = Extremely feasible.

Data analysis used the Group WisdomTM software (Concept Systems, Inc., Ithaca, NY) (30). A similarity matrix was created to identify how often statements were sorted together in the same cluster. Through multidimensional scaling, the similarity matrix was used to generate a two-dimensional “point map” of each statement to visually represent the sorted data, with statements sorted together more often placed closer on the map. A stress value statistic was generated, which indicated how well the 2-dimensional point map represented the sorted data. Hierarchical cluster analysis was then used to aggregate the point coordinates into clusters which contained similar statements. A bridging value was generated for each statement, indicating whether it was sorted with other statements nearby (referred to as anchoring), or sorted with others across a larger area of the map (referred to as bridging). Lower bridging values (range 0–1) indicated how closely individual statements were related and extent of agreement in sorting across participants. The number of clusters were initially chosen by the researchers and iterations were dependent on discussions with students. Four quadrants “Go-Zone” maps were also generated, which provided a visual display of an XY graph and was divided into quadrants above and below the mean ratings for importance and feasibility. The statements located in the upper right of the figure were considered to be rated above average for both importance and feasibility.

Permission and ethical approval were received from the Ministry of Education (MoE) in Jeddah (KSA). The project was also approved by King's College London Ethics Committee (*REC Reference Number 3727*) London, UK.

## Results

### Perspectives of Students on the Factors That Influence Dietary and Physical Activity Habits

Students used 20 photographs to aid the generation of 42 statements, 25 in relation to dietary factors and 17 in relation to physical activity. Table 1 shows clusters for dietary habits and physical activity, average bridging values and average ratings for importance and feasibility for each cluster, statements that were rated relatively high for both importance and feasibility (upper right quadrant of the Go-Zone map) and exemplar photographs taken by students to generate statements. Supplementary Table 1 shows all statements by cluster, along with the bridging values and ratings for each statement.

**Table 1:** Students' perspectives on factors influencing their dietary habits and physical activity: average bridging values and average ratings for importance and feasibility for each cluster, statements that were rated relatively high for both importance and feasibility (upper right quadrant of the Go-Zone map) and exemplar photographs taken by students to generate statements.

**Cluster name**	**Bridging value**	Average importance score^*^	Average feasibility score^*^	Example of statements rated as most important and feasible, with exemplar pictures that were taken by students^**^	
**Influences on dietary habits**					
Role of Government	0.07	4.19	4.17	4-Healthy food is limited in the mall 5-Healthy meal are high 18-Reduce the cost of healthy food 23-More salad in fast-food restaurants	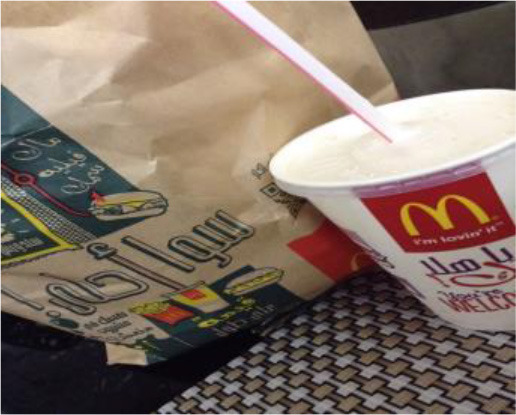
School Environment	0.38	3.79	3.82	9-Chocolates are available in the canteen 13-The school environment needs to be supportive 20-The school environment influences behaviors 21-Offer fruit and vegetables in the canteen 22-Provide vending machine for fruit and vegetables	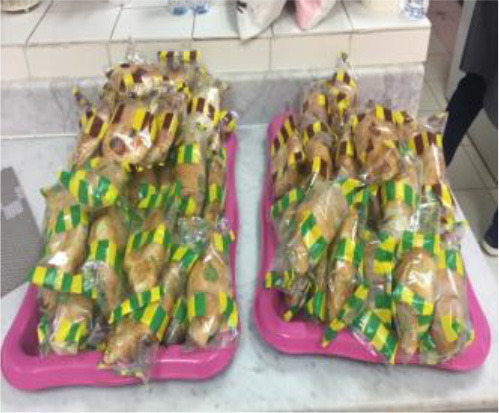
Home Environment	0.53	3.99	4.02	15-The food at home should be healthy 16-Salads are healthy 17-Eat fruit every day 25-Changes in behavior are influenced by family	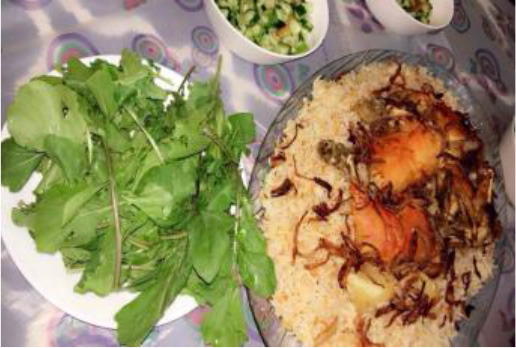
Retail Environment	0.66	3.87	2.43	No statement rated as most important and feasible	
**Influences on physical activity**					
Role of Government	0.09	4.07	3.98	1-Gym prices are high 2-Lack of female gyms 7-Unhealthy food is available at gyms 12-Lack of attractive activities in the community 16-Provide gyms and walking areas 20-Provide free indoor gyms	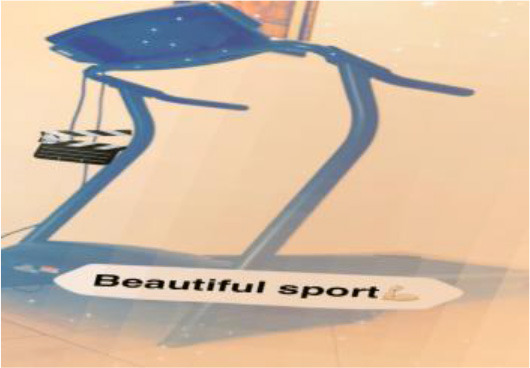
Cultural practices	0.76	3.62	3.17	No statement rated as most important and feasible	
School Environment	0.86	3.57	3.29	11-Lack of attractive activities at school, 15- Physical activity as a part of the school curriculum	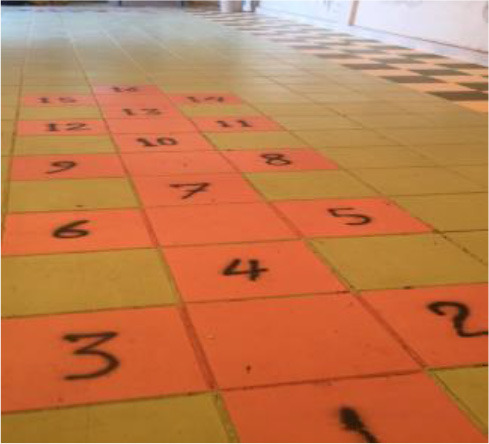

* *Score out of 5*.

** *Derived from the upper right quadrant of the Go-Zone map*.

**Figure 1:**
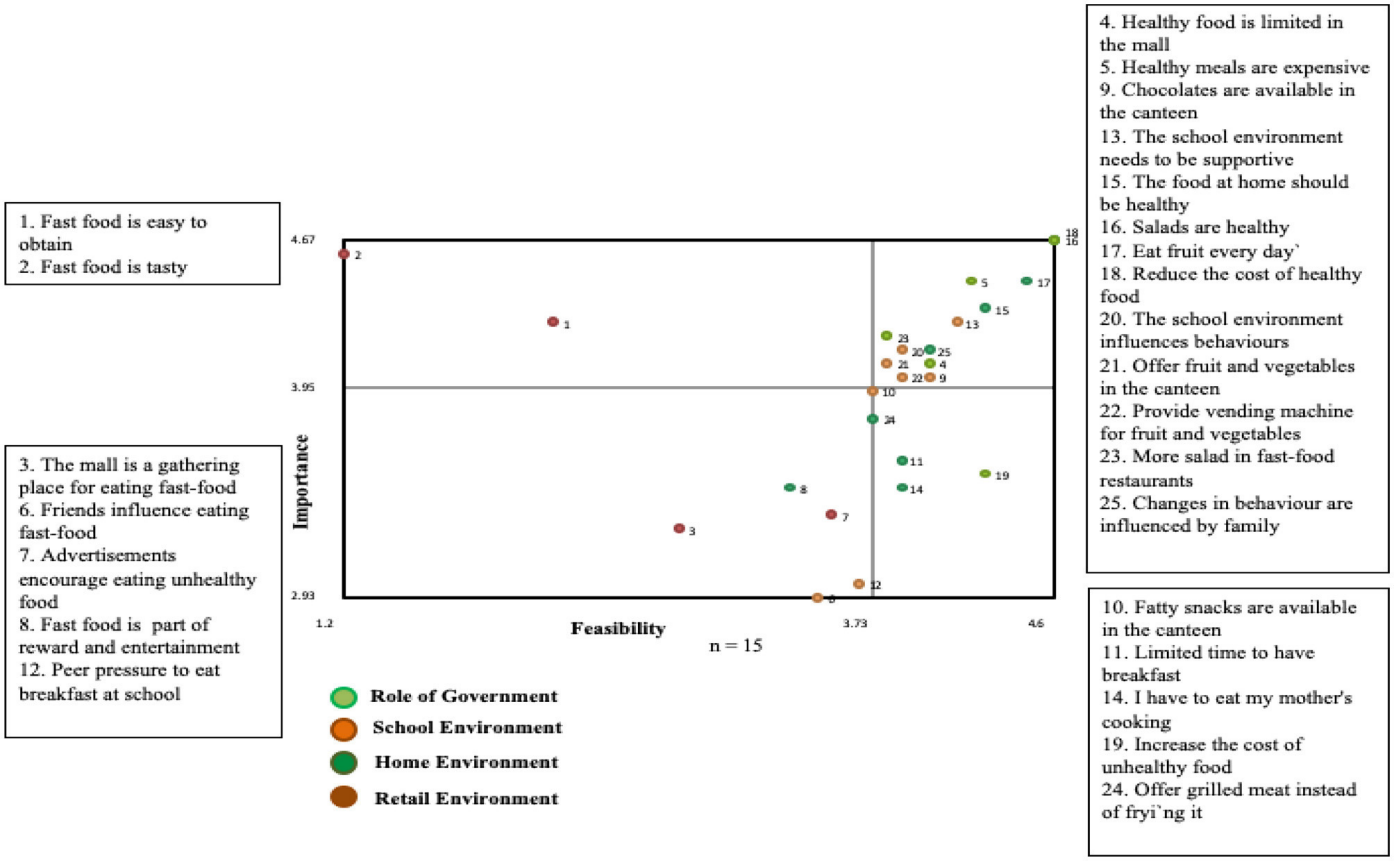
Go-zone map based on students' perspectives on the factors that influence their dietary habit.

Five clusters were identified in relation to dietary habits—“Role of Government,” “School Environment,” “Home Environment,” and “Retail of Fast Foods.” Average bridging values for clusters were lowest for “Role of Government” (0.07) and highest for “Retail Environment” (0.66). The “Role of Government” had the highest ratings for both importance (4.19) and feasibility of change (4.17) and “Retail of fast foods” had the lowest rating for feasibility (2.43). Overall these results reflected that the role of government was felt to be an important determinant of feasible action across students. The clusters “Home Environment” and “School Environment” had high average ratings for both importance and feasibility. The bridging values, however, were high for specific statements which indicated a lack agreement in clustering of these statements across students. For example, the cluster “School Environment” contained statements with high bridging values such as the influence of friends on eating fast foods (bridging value 0.92), peer pressure to eat breakfast at school (0.64), and vending machines for fruits and vegetables (0.84). Figure 1 shows the Go-Zone map based on students' ratings for importance and feasibility for change for all statements. Approximately half of the statements were rated above average for both importance and feasibility (upper right quadrant). Four of the statements from the cluster “Role of Government” were included and about half of the statements from the “School Environment” and “Home Environment” were included.

The statements that were rated highest on both importance and feasibility in the cluster “Role of Government” indicated an awareness of issues related to availability (e.g. healthy foods limited in malls, salad in fast food restaurants) and accessibility (e.g. costs) of health foods. “School Environment” issues generally referred to availability of foods in the canteen and the “Home Environment” to the family playing an important role in promoting healthy eating habits. Noteworthy is that the statements from the cluster “Retail Environment” were rated below average for both importance and feasibility (lower left quadrant). These referred to the mall being a gathering place for eating fast food.

Three clusters were identified in relation to physical activity—“Role of Government,” “Cultural Practices” and “School Environment.” Average bridging values for the clusters were lowest for “Role of Government” (0.10) and highest for “School Environment” (0.86). The “Role of Government” had the highest ratings for both importance (4.07) and feasibility of change (3.98) and “Cultural Practices” had the lowest rating for feasibility (3.17). As with dietary factors, the role of government was perceived to be an important determinant of feasible action. All statements within this cluster had low bridging values which indicated strong correspondence across students in how they clustered the statements. In contrast, the statements in the other two clusters had high bridging values. The majority of the statements that were in the upper right quadrant (Table 1; Supplementary Figure 1) were from the cluster “Role of Government,” and referred to structural issues such as the lack of female only gyms, cost of membership, lack of transport and the need for walking areas. “School Environment” issues that were rated high referred to the need for inclusion of physical activity in the curriculum. A notable feature of the Go-Zone map was that the intersection of the importance and feasibility axes was above ratings of 3 (out of 5) and many of the statements that were not in the go-zone were around the intersection. For example, issues related to transport to the gym, and use of technology, were present in the quadrant for low feasibility, low importance quadrant, whereas these were rated above 3 for both importance and feasibility. Cultural practices were rated lowest for both feasibility and importance and were not part of the Go-Zone.

### Perspectives of Adults on the Factors That Influence Adolescent Obesity and on Feasible Priorities for the Content of a School-Based Programme

Adults generated 77 statements, 35 in relation to factors influencing adolescent obesity and 42 in relation to the content of school-based programmes. Table 2 shows clusters, average bridging values and ratings for importance and feasibility for each cluster, and the statements that were rated high for both importance and feasibility (upper right quadrant of the Go-Zone map). Supplementary Table 2 shows all statements by cluster, along with the bridging values and ratings for the clusters and statements.

**Table 2:** Adults' perspectives on factors that influence adolescent obesity and on feasible priorities for the content of school-based programmes: average bridging values and average ratings for importance and feasibility for each cluster, statements that were rated relatively high for both importance and feasibility (upper right quadrant of the Go-Zone map).

**Cluster solution**	**Bridging value**	Average important score^*^	Average feasibility score^*^	Statements rated as most important and feasible^**^
**Perspectives of adults on the factors that influence adolescent obesity**				
1-Community	0.42	4.02	2.95	No statement rated as most importance and feasible
2-Biology	0.68	3.42	2.68	No statement rated as most importance and feasible
3-Lifestyle	0.13	3.86	3.6	4-High-calorie and high-carbohydrate intake 8-Fizzy drinks and high-calorie energy drinks 15-Sedentary lifestyles associated with electronic devices
4-School environments	0.22	4.11	3.89	14-Awareness of physical activity,19- Fast-food restaurants 27-Drink water at school 29-Insufficient physical activity 34-Family awareness 35-Check-ups for general health
5-Home environment	0.03	3.94	3.90	20-Eat a healthy diet 24-Eat fruit and vegetables 28-Follow Islamic rules in diet habits 30-Snacks in front of the TV 31-Time spent watching TV 33-Awareness of sleep as a healthy behavior
**Perspectives of adults on feasible priorities for the content of a school-based programme**				
1-Ministry of Education Support	0.07	4.03	3.98	1-Offer healthy food in the canteen 2-Remove chocolate and sweets 7-Have a dietitian to advice the canteen 8-Establish connection between health center and the canteen 13-Attractive healthy meals in school 29-Nutritionists should be part of the programme 32-Lack of healthy and suitable diets 33-Lack of specialist school meal supervisors
2-Public health programmes	0.46	3.79	3.84	3- Awareness of unhealthy foods such as fast foods
3-Schools Environment	0.14	3.83	3.92	4-Educate children about healthy food 5-Educate children about obesity 6-Offer practice programme 26-Health guides in schools
4-Wider environmental influences	0.7	3.96	3.82	12-Encourage healthy breakfasts 14-Encourage walking 30-Promote family responsibility 38-Lack of family knowledge about obesity

* *Score out of 5*.

** *Derived from the upper right quadrant of the Go-Zone map. The number preceding the statement corresponds to the number of the statement on the Go-Zone map*.

Five clusters were generated in relation to factors influencing adolescent obesity. These were “Home environment,” “Lifestyle,” “School Environment,” “Community” and “Biology” (Table 2). There was a high level of agreement across adults regarding how they classified statements in the first three clusters, reflected by the low bridging values. Average cluster ratings ranged from 3.42 to 4.02 for importance and 2.68 to 3.90 for feasibility. “School Environment” had the highest average cluster ratings for importance, and both “School Environment” and “Home Environment” for feasibility. Of the 15 statements in the Go-Zone (Supplementary Figure 2), 12 were from the “Home Environment” and “School Environment” clusters that rated high for both importance and feasibility. For “Home Environment,” the issues raised related to healthy and regular meals, time spent watching TV and the importance of sleep. For “School Environment,” the issues raised referred to insufficient physical activity, importance of drinking water and raising family awareness. Action to reduce high calorie foods and drinks and use of electronic devices were also included in the Go-Zone, captured under the cluster “Lifestyle.” “Biology” had the lowest ratings for importance and feasibility. It is also worth mentioning that although issues such as eating fast foods, dining out and advertisements of convenience foods were felt to be highly important, they were rated relatively low for feasibility (upper left quadrant of the Go-Zone map, Supplementary Figure 2).

**Figure 2:**
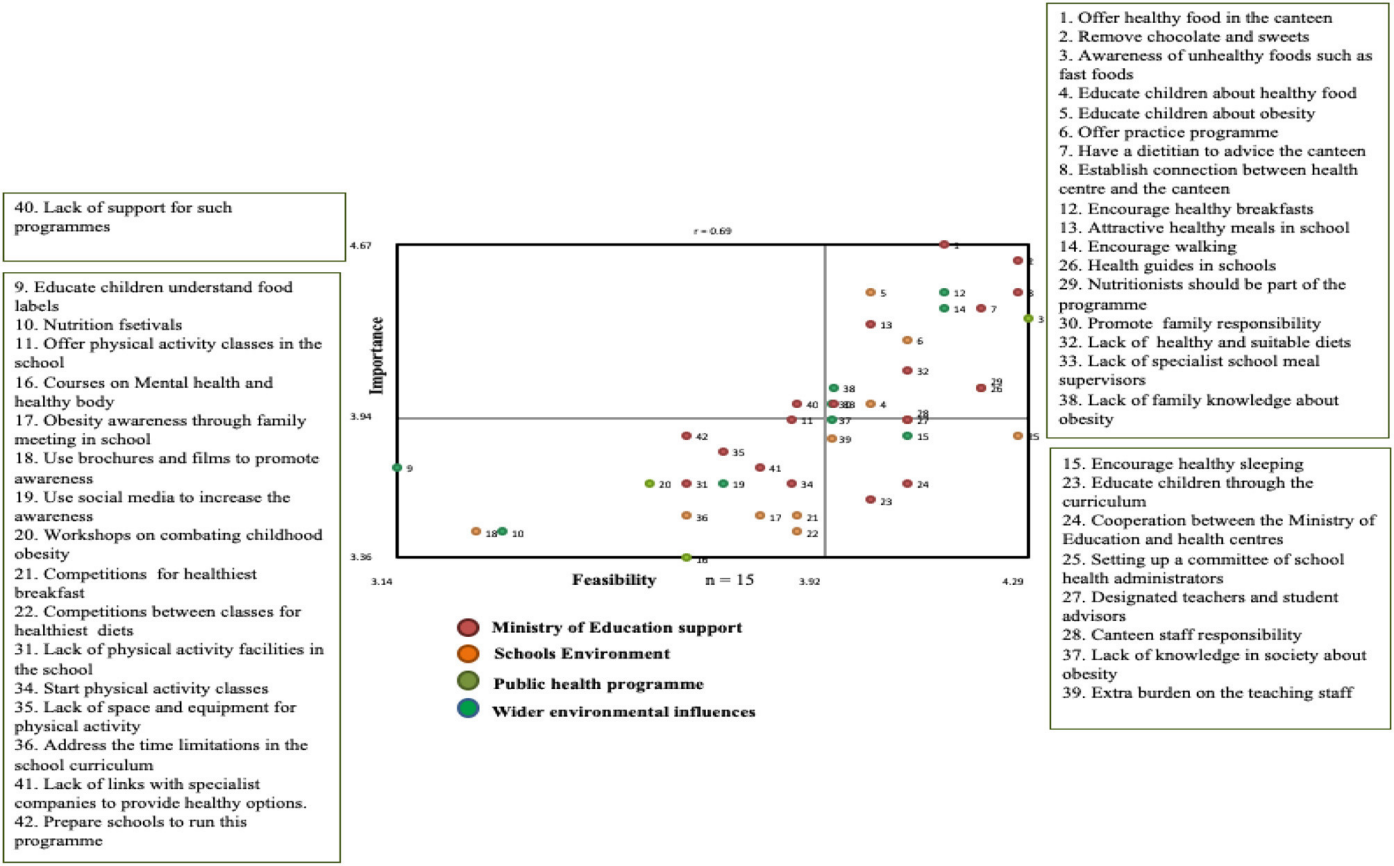
Go-zone map based on adults' perspectives on the content of a school-based programme.

Four clusters were generated in relation to the content of a school programme. These were “Ministry of Education Support,” “School Environment,” “Public health programmes,” and “Wider environmental influences” (Table 2). The clusters “Ministry of Education Support” (0.07) and “School Environment” (0.14) had the lowest overall bridging values. The Go-Zone (Figure 2) included system related issues raised under the cluster “Ministry of Education Support,” such as availability of dietitians, nutritionists, specialist school meal supervisors, and school connections with the health centers. Education activities as well as having students as health guides were raised under “School Environment.” In addition, the Go-Zone included issues related to encouraging healthy breakfasts and walking as well as engaging families, which were from the cluster “Wider environmental influences.” The intersection of the axes reflected ratings to be relatively lower for importance than for feasibility. Several statements were in the lower left quadrant of the Go-Zone map plot and were rated to be more feasible than important. These included activities such as adapting the school curriculum, and using competitions, workshops and social media to promote healthy eating and physical activity.

## Discussion

Using group concept mapping, perspectives from students and those responsible for planning and provision of intermediate school education services provided valuable insights into the factors influencing adolescent obesity and the potential content of a school-based nutrition intervention programme. Students identified several factors at different social-ecological levels, including the role of government, family life, school and wider environments and traditions that influence their dietary and physical activity behaviors. These factors largely resonated in the perspectives of the school and Ministry of Education staff on factors influencing adolescent obesity. Several focused suggestions were given for a school-based programme by both students and adults, with the support of Government felt to be important and feasible. This study adds to a scant evidence base on the use of participatory approaches with schools in the Gulf region. Both students and adults engaged with enthusiasm to express their perspectives on obesity-related health behaviors based on their different experiences, expertise and perception of needs to address the complex issue of prevention.

There was consistent consensus across students and adults that the support of government was important and feasible for the prevention of obesity. Regulatory interventions are essential to drive sustainable environmental and social changes to reduce obesity (31). The recent initiatives in Saudi relating to carbonated drinks and energy labeling on menus could have created a context of heightened awareness for the need for obesity prevention initiatives (6, 32). But it is also worth considering the changing social and economic landscape of Saudi as it prepares for a future in which oil resources will play a far less significant role in the economy than has historically been the case. Young people are growing up in an interesting transitional context with the potential expansion of entertainment and tourism industries, reforms to the education system as well as increasing use of social media (33). Saudi's Vision 2030 aims to activate the role of youths in society, with Youth Councils being a mechanism for their involvement in decision-making, as well as maintaining loyalty and belonging to the Kingdom (20, 21, 34). The significance of youths in Saudi cannot be underestimated as they comprise 70% of Saudi society. The articulate voices of Saudi adolescents in the current study reflected a unique scenario of adolescents confidently driving their change agenda to protect their health in the context of a societal shift in exposures and belief that government will support their request.

The role of home environments in preventing obesity was also emphasized across students and adults. School and Ministry of Education staff felt that families had a responsibility to limit use of digital devices, time spent watching TV, and consumption of high calorie and sugary foods. A systematic review on the influence of home environment on childhood obesity have shown parenting style and practices (i.e., pressure to eat), knowledge and perceptions about child weight, TV in a child's bedroom, physical activity behavior and shortage of sleep to be associated with child weight status and health behaviors, and that there is a strong interplay between these home environment factors as they reinforce each other (35–37). Involving parents in an obesity prevention programme is therefore essential and was consistently reported by students and adults to be important for establishing healthy lifestyles.

Students and adults identified school environments as targets for feasible interventions. The removal of unhealthy food (high fat and sugary foods in particular) in the school canteen was seen as an important and feasible to change. Provision of healthy food in schools can increase choice and consumption of healthy foods in the school environment (38, 39), and also affect healthy food (fruit and vegetables) purchasing and consumption outside the school environment (40–42). For example, the Child and Adolescent Trial for Cardiovascular Health (CATCH) study showed that modifying food provision in schools and implementing a health curriculum led to a significantly higher decrease in the percentage of energy intake from fat (38.7% to 31.9%, *p* = < 0.001) in the diets of the students in the intervention group compared with those in the control group (43). An intervention that makes a change to the school environment, such as how food is served and presented, might also influence children's behavior by reducing weight gain, facilitating healthy eating choices and creating a supportive school environment. However, without local level implementation support, government school nutrition policies and school systems are unlikely to yield improvement in the school food environment.

### Strengths and Limitations

To our knowledge, this is the first concept mapping study carried out with planners and providers of education services and students in Saudi Arabia to examine adolescent obesity prevention, and which provides significant insights into how students' voices can be integrated into key decision-making dialogues. Strengthened by the use of their own photographs of their environments, the method embedded adolescents in the research from the onset, giving a sense of ownership and contribution. It also drew on the considerations in both individual and group-oriented activities (28). Their experiences and judgments on sorting and ratings of importance and feasibility for change improved the validity of the entire analysis, including the statistical analysis. This mixed approach is a substantially stronger methodological approach than using only a quantitative or qualitative approach for understanding a complex phenomenon, allowing the exploration of multiple themes and their inter-relationships (44). There are, however, several limitations. The study was based on 15 students in two schools and 25 adults, and the findings may not be generalizable. Insufficient number of mothers participated and although there was strong agreement on the feasible actions Go-Zone, a larger and socio-economically diverse sample of parents may have raised different issues (e.g. time restrictions among working mothers to prepare freshly cooked meals, deferring such responsibilities to careers etc.) (45, 46). Concept mapping methodology shares limitations associated with issues around the generalizability of the findings due to non-random sampling, dependence on the capabilities of the participant, and participants not having contributed equally in statements generation sessions. For cultural reasons, fathers were not included in the concept mapping workshops and we were unable to conduct the study with boys as it would have required a male researcher. That was clearly a major gap that should be addressed in future studies.

## Conclusion

Students and school and MoE staff jointly agreed that a canteen-based intervention was important and feasible to improve dietary habits and thus help to prevent obesity among adolescent girls. This was the first time a participatory approach was with students for intervention development in Saudi Arabia. Students and adults engaged with the entire process with enthusiasm and respect, which signaled that a co-development approach may have value to improve school food environments in Saudi Arabia.

## Data Availability Statement

The original contributions presented in the study are included in the article/Supplementary Material, further inquiries can be directed to the corresponding author.

## Ethics Statement

Written informed consent was obtained from the individual(s), and minor(s)' legal guardian/next of kin.

## Author Contributions

MA conceived the idea as part of her doctoral thesis, conducted all workshops, and led the initial draft. SH and MO'K were PhD supervisors of MA. All authors participated in the discussions that led to the design of the study, contributed substantially to the development of the study, made critical revisions to the manuscript, and provided final approval of the version to publish.

## Funding

SH was funded by MR/N015959/1, MR/S009035/1, and MR/R022739/1. MO'K was funded by MR/S009035/1 and MR/R022739/1. MA was funded by Saudi Scholarship.

## Conflict of Interest

The authors declare that the research was conducted in the absence of any commercial or financial relationships that could be construed as a potential conflict of interest.

## Publisher's Note

All claims expressed in this article are solely those of the authors and do not necessarily represent those of their affiliated organizations, or those of the publisher, the editors and the reviewers. Any product that may be evaluated in this article, or claim that may be made by its manufacturer, is not guaranteed or endorsed by the publisher.

## References

[1] Almughamisi M. The Co-development of a School-Based Nutrition Intervention to Prevent Childhood Obesity in Jeddah, Saudi Arabia. London: King's College London (2021).

[2] Musaiger AO. Overweight and obesity in eastern mediterranean region: prevalence and possible causes. J Obes. (2011) 2011:407237. 10.1155/2011/407237 21941635 PMC3175401

[3] Templin T Cravo Oliveira Hashiguchi T Thomson B Dieleman J Bendavid E. The overweight and obesity transition from the wealthy to the poor in low-and middle-income countries: a survey of household data from 103 countries. PLoS Med. (2019) 16:e1002968. 10.1371/journal.pmed.1002968 31774821 PMC6880978

[4] Mahfouz AA Abdelmoneim I Khan MY Daffalla AA Diab MM Al-Gelban KS. Obesity and related behaviors among adolescent school boys in Abha City, Southwestern Saudi Arabia. J Trop Pediatr. (2007) 54:120–4. 10.1093/tropej/fmm089 18039676

[5] Al Alwan I Al Fattani A Longford N. The effect of parental socioeconomic class on children's body mass indices. J Clin Res Pediatr Endocrinol. (2013) 5:110–5. 10.4274/Jcrpe.898 23748064 PMC3701916

[6] Alkhaldy AA Taha DS Alsahafi SE Naaman RK Alkhalaf MM. Response of the public and restaurant owners to the mandatory menu energy-labelling implementation in restaurants in Saudi Arabia. Public Health Nutr. (2020) 23:3435–47. 10.1017/S1368980020000245 32450940 PMC10200400

[7] Yuksel HS Sahin FN Maksimovic N Drid P Bianco A. School-based intervention programs for preventing obesity and promoting physical activity and fitness: a systematic review. Int J Environ Res Public Health. (2020) 17:347. 10.3390/ijerph17010347 31947891 PMC6981629

[8] Madhi ST Barrientos A. Saudisation and employment in Saudi Arabia. Career Dev Int. (2003) 8:70–7. 10.1108/1362043031046547129214078

[9] The Word Bank. GDP Growth (Annual %) - Saudi Arabia. (2019). Available online at: https://data.worldbank.org/indicator/NY.GDP.MKTP.KD.ZG?end=2019&locations=SA&start=1969&view=chart (accessed January, 2020).

[10] Popkin BM Adair LS Ng SW. Global nutrition transition and the pandemic of obesity in developing countries. Nutr Rev. (2012) 70:3–21. 10.1111/j.1753-4887.2011.00456.x 22221213 PMC3257829

[11] World Health Organization. Noncommunicable Diseases Country Profiles 2014. (2014). Available online at: https://www.who.int/nmh/publications/ncd-profiles-2014/en/ (accessed March, 2020).

[12] Aboul Enein BH Bernstein J Neary A. Dietary transition and obesity in selected Arabic-speaking countries: a review of the current evidence. East Mediterr Health J. (2016) 22:763–70. 10.26719/2016.22.10.763 28134430

[13] S, Gao, tatistics. Population of the Adminstrative Region of Makkah. Available online at: https://www.stats.gov.sa/sites/default/files/ar-makkah.pdf (accessed June, 2020).

[14] Memish ZA El Bcheraoui C Tuffaha M Robinson M Daoud F Jaber S. Peer reviewed: obesity and associated; factors—Kingdom of Saudi Arabia, 2013. Prev Chronic Dis. (2014) 11:E174. 10.5888/pcd11.140236 25299980 PMC4193060

[15] Samara A Andersen PT Aro AR. Health promotion and obesity in the Arab Gulf states: challenges and good practices. J Obes. (2019) 2019:4756260. 10.1155/2019/4756260 31281673 PMC6590587

[16] Middleton G Evans AB Keegan R Bishop D Evans D. The Importance of Parents and Teachers as Stakeholders in School-Based Healthy Eating Programs. 2014. Health Education: Parental and Educators' Perspectives, Current Practices and Needs Assessment. Health Care Issues, Costs and Access. New York, NY: NOVA Science Publishers. Available online at: http://eprints.lincoln.ac.uk/11965/ (accessed June, 2020).

[17] Farr MDR Davies P Bagnall D Brangan E Andrews H. (2020). A Map of Resources for Co-producing Research in Health and Social Care. Bristol: National Institute for Health Research (NIHR) ARC West and People in Health West of England. University of Bristol and University of West of England (2020).

[18] Norström AV Cvitanovic C Löf MF West S Wyborn C Balvanera P. Principles for knowledge co-production in sustainability research. Nat Sustain. (2020) 3:182–90. 10.1038/s41893-019-0448-234745368

[19] Griebler U Rojatz D Simovska V Forster R. Effects of student participation in school health promotion: a systematic review. Health Promot Int. (2017) 32:195–206. 10.1093/heapro/dat090 24395957

[20] Alkohaiz MA. bin Shalhoub HA. Structuring youth councils in Saudi Arabia: a forecast study. Acad J Interdiscip Stud. (2021) 10:96. 10.36941/ajis-2021-0009

[21] Alshanbri AM. Empowering positive youth development in Saudi Arabia: youth civic engagement, Agenda setting and policy formulation (Electronic thesis). University of Arkansas, Fayetteville, AR, United States (2014). 10.1186/s12961-017-0231-7

[22] Kane M Trochim WM. Concept Mapping for Planning and Evaluation. Thousand Oaks, CA: Sage Publications (2007). 10.4135/9781412983730

[23] Wutzke S Roberts N Willis C Best A Wilson A Trochim W. Setting strategy for system change: using concept mapping to prioritise national action for chronic disease prevention. Health Res Policy Syst. (2017) 15:69. 28784177 10.1186/s12961-017-0231-7 PMC5547536

[24] Trochim WM. An introduction to concept mapping for planning and evaluation. Eval Program Plann. (1989) 12:1–16. 10.1016/0149-7189(89)90016-5

[25] Wang C Burris MA. Photovoice: concept, methodology, and use for participatory needs assessment. Health Educ Behav. (1997) 24:369–87. 10.1177/109019819702400309 9158980

[26] Schophuizen M Kreijns K Stoyanov S Kalz M. Eliciting the challenges and opportunities organizations face when delivering open online education: a group-concept mapping study. Internet High Educ. (2018) 36:1–12. 10.1016/j.iheduc.2017.08.002

[27] Freeman LA Jessup LM. The power and benefits of concept mapping: measuring use, usefulness, ease of use, and satisfaction. Int J Sci Educ. (2004) 26:151–69. 10.1080/0950069032000097361

[28] Burke JG O'Campo P Peak GL Gielen AC McDonnell KA Trochim WM. An introduction to concept mapping as a participatory public health research method. Qual Health Res. (2005) 15:1392–410. 10.1177/1049732305278876 16263919

[29] Haque N Rosas S. Concept mapping of photovoices: sequencing and integrating methods to understand immigrants' perceptions of neighborhood influences on health. Fam Community Health. (2010) 33:193–206. 10.1097/FCH.0b013e3181e4bbf0 20531100

[30] Trochim WM Cook JA Setze RJ. Using concept mapping to develop a conceptual framework of staff's views of a supported employment program for individuals with severe mental illness. J Consult Clin Psychol. (1994) 62:766. 10.1037/0022-006X.62.4.766 7962880

[31] Swinburn BA. Obesity prevention: the role of policies, laws and regulations. Aust N Z Health Policy. (2008) 5:12. 10.1186/1743-8462-5-12 18534000 PMC2440375

[32] Alsukait R Wilde P Bleich SN Singh G Folta SC. Evaluating Saudi Arabia's 50% carbonated drink excise tax: changes in prices and volume sales. Econ Hum Biol. (2020) 38:100868. 10.1016/j.ehb.2020.100868 32302767

[33] Makki E Chang L-C. Understanding the effects of social media and mobile usage on e-commerce: an exploratory study in Saudi Arabia. Int Manag Rev. (2015) 11:98–109.

[34] Alkathiri ESA. The Relationship Between Saudi Citizen Perceptions Of Vision 2030 And Saudi Government Social Media Use To Promote Vision 2030. Grand Forks, ND: The University of North Dakota (2020).

[35] Bates C Buscemi J Nicholson L Cory M Jagpal A Bohnert A. Links between the organization of the family home environment and child obesity: a systematic review. Obes Rev. (2018) 19:716–27. 10.1111/obr.12662 29520946

[36] Sahoo K Sahoo B Choudhury AK Sofi NY Kumar R Bhadoria AS. Childhood obesity: causes and consequences. J Fam Med Primary Care. (2015) 4:187. 10.4103/2249-4863.154628 25949965 PMC4408699

[37] Barnett TA Kelly AS Young DR Perry CK Pratt CA Edwards NM. Sedentary behaviors in today's youth: approaches to the prevention and management of childhood obesity: a scientific statement from the American Heart Association. Circulation. (2018) 138:e142–e59. 10.1161/CIR.0000000000000591 30354382

[38] Upton D Upton P Taylor C. Increasing children's lunchtime consumption of fruit and vegetables: an evaluation of the Food Dudes programme. Public Health Nutr. (2013) 16:1066–72. 10.1017/S1368980012004612 23067425 PMC10271278

[39] Olsho LE Klerman JA Ritchie L Wakimoto P Webb KL Bartlett S. Increasing child fruit and vegetable intake: findings from the US Department of Agriculture Fresh Fruit and Vegetable Program. J Acad Nutr Diet. (2015) 115:1283–90. 10.1016/j.jand.2014.12.026 25746429

[40] Ganann R Fitzpatrick-Lewis D Ciliska D Peirson LJ Warren RL Fieldhouse P. Enhancing nutritional environments through access to fruit and vegetables in schools and homes among children and youth: a systematic review. BMC Res Notes. (2014) 7:1–13. 10.1186/1756-0500-7-422 24996963 PMC4114435

[41] Ismail MR Seabrook JA Gilliland JA. Outcome evaluation of fruit and vegetables distribution interventions in schools: a systematic review and meta-analysis. Public Health Nutr. (2021) 24:4693–705. 10.1017/S1368980021001683 33866997 PMC10195380

[42] Ohri-Vachaspati P Dachenhaus E Gruner J Mollner K Hekler EB Todd M. Fresh fruit and vegetable program and requests for fruits and vegetables outside school settings. J Acad Nutr Diet. (2018) 118:1408–16. 10.1016/j.jand.2017.10.013 29325891

[43] Luepker RV Perry CL McKinlay SM Nader PR Parcel GS Stone EJ. Outcomes of a field trial to improve children's dietary patterns and physical activity: the Child and Adolescent Trial for Cardiovascular Health (CATCH). Jama. (1996) 275:768–76. 10.1001/jama.1996.03530340032026 8598593

[44] Jackson KM Trochim WM. Concept mapping as an alternative approach for the analysis of open-ended survey responses. Organ Res Methods. (2002) 5:307–36. 10.1177/109442802237114

[45] Tobío C. Working and mothering-women's strategies in Spain. Eur Soc. (2001) 3:339–71. 10.1080/14616690120079369

[46] Sweet S Moen P. Integrating educational careers in work and family: women's return to school and family life quality. Commun Work Fam. (2007) 10:231–50. 10.1080/13668800701270166

