# Supplementary material for: Adolescent Obesity Prevention in Saudi Arabia: Co-identifying Actionable Priorities for Interventions
Source: Front Public Health. 2022 May 10;10:863765. doi: 10.3389/fpubh.2022.863765 (PMC9128526; doi:10.3389/fpubh.2022.863765)
Supplement: Supplementary file 1 [file Data_Sheet_1.pdf]

Online Table 1. Dietary habits and physical activity: students generated statements within clusters, and bridging values and average ratings for importance and feasibility.

Cluster		Statements	Bridging value	Importance rating	Feasibility rating
**Influences on dietary habits**					
**1- Retail Environment**			**0.66**	**3.87**	**2.43**
	1	Fast food is easy to obtain	0.63	4.27	2.2
	2	Fast food is tasty	0.76	4.6	1.2
	3	The mall is a gathering place for eating fast-food	1	3.27	2.8
	7	Advertisements encourage eating unhealthy food	0.26	3.33	3.53
**2- Role Government**			**0.07**	**4.19**	**4.17**
	4	Healthy food is limited in the mall	0	4.07	4
	5	Healthy meals are expensive	0.06	4.47	4.2
	18	Reduce the cost of healthy food	0	4.67	4.6
	19	Increase the cost of unhealthy food	0	3.53	4.27
	23	More salad in fast-food restaurants	0.31	4.2	3.8
**3 -Schools Environment**			**0.38**	**3.79**	**3.82**
	6	Friends influence eating fast-food	0.92	2.93	3.47
	9	Chocolates are available in the canteen	0.13	4	4
	10	Fatty snacks are available in the canteen	0.13	3.93	3.73
	12	Peer pressure to eat breakfast at school	0.64	3	3.67
	13	The school environment needs to be supportive	0.13	4.27	4.13
	20	The school environment influences behaviours	0.13	4.13	3.87
	21	Offer fruit and vegetables in the canteen	0.13	4.07	3.8
	22	Provide vending machine for fruit and vegetables	0.84	4	3.87
**4 -Home Environment**			**0.53**	**3.99**	**4.02**
	8	Fast food is part of reward and entertainment	0.35	3.47	3.33
	11	Limited time to have breakfast	0.67	3.6	3.87
	14	I have to eat my mother's cooking	0.35	3.47	3.87
	15	The food at home should be healthy	0.35	4.33	4.27
	16	Salads are healthy	0.69	4.67	4.6
	17	Eat fruit every day	0.57	4.47	4.47
	24	Offer grilled meat instead of frying it	0.48	3.8	3.73
	25	Changes in behaviour are influenced by family	0.82	4.13	4

Influences on Physical activity

**1 – Roles of Government**		**0.1**	**4.07**	**3.98**
1	Gym prices are high	0.09	4.47	3.8
2	Lack of female gyms	0	3.8	4.2
3	Gyms are far from home	0.3	3.73	3.47
4	Lack of transport to get to the gym	0.25	4.07	3.33
7	Unhealthy food is available at gyms	0.16	4.2	4.07
12	Lack of attractive activities in the community	0.04	4.33	4.27
13	Lack of fun places	0.04	3.33	4.13
16	Provide gyms and walking areas	0	4.53	4.53
17	Provide free indoor gyms	0	4.13	4
**2- Schools Environment**		**0.86**	**3.57**	**3.29**
5	Too far to walk to school	1	3.8	3.07
9	Hot weather is a barrier to outdoor physical activity	0.84	2.47	1.2
11	Lack of attractive activities at school	0.79	4.13	4.2
14	Extra homework is given on weekdays.	0.92	3	3.4
15	Physical activity as a part of the school curriculum	0.77	4.47	4.6
**3 -Cultural Practices**		**0.76**	**3.39**	**3.16**
6	Outdoor physical activity is difficult with hijabs	0.74	3.4	3
8	Culture do not allow us to go out every day	0.65	2.93	3.13
10	Boring routines encourage use of technology	0.9	3.86	3.33

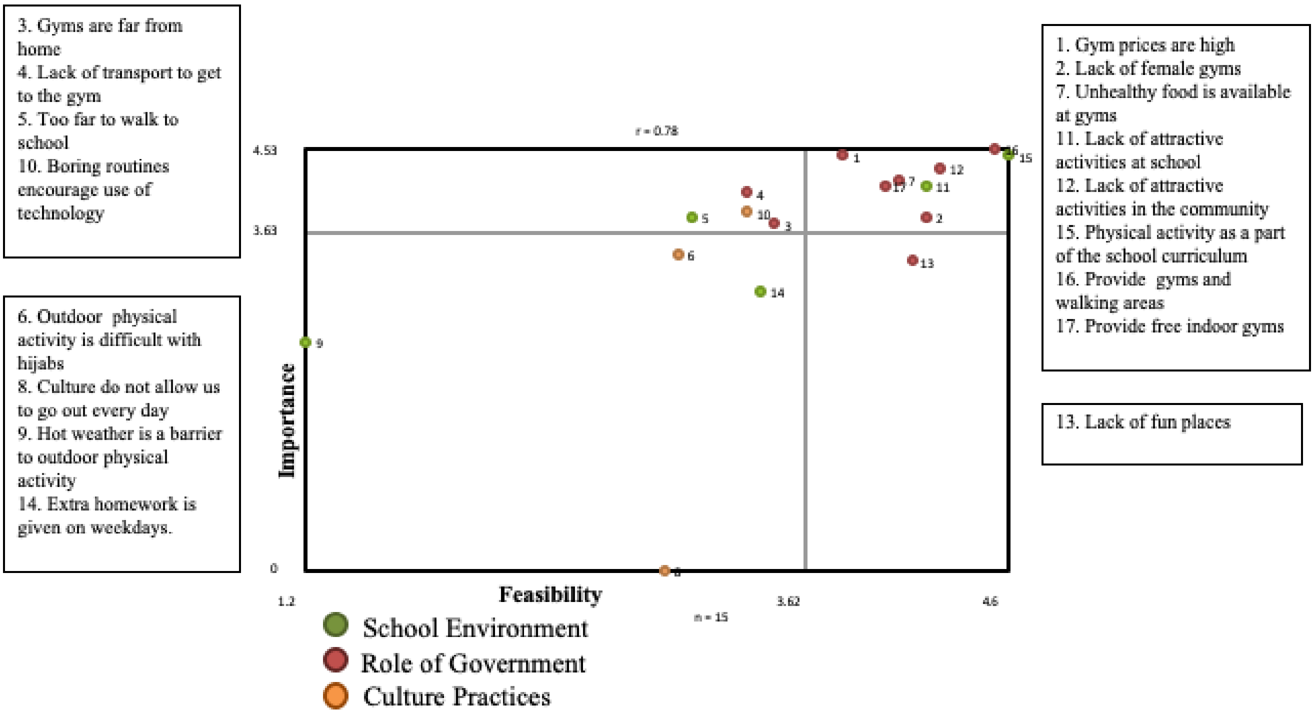

Online Figure 1. Go-zone map based on students' perspectives on the factors that influence their physical activity

Online Table 2 . Factors that influence adolescent obesity and feasible priorities for the content of a school-based programme: adult generated statements within clusters, and bridging values and average ratings for importance and feasibility.

Cluster		Statements	Bridging value	Average importance score	Average feasibility score
**Influences on adolescent obesity**					
**1- Community**			**0.42**	**4.02**	**2.95**
	1	Eat fast-food	0.41	4.33	2.5
	3	Large portion sizes	0.36	3.8	3.2
	5	Misinformation in the media	0.54	3.73	2.7
	6	Advertisements of convenience food	0.38	4.21	3.2
**2- Biology**			**0.68**	**3.42**	**2.68**
	16	Genetic factors	0.52	3.2	2
	17	Hormonal changes	0.51	3.33	2.2
	32	Emotional Health	1	3.73	3.7
**3 -Lifestyle**			**0.13**	**3.86**	**3.6**
	2	Frequency of dining out	0.1	3.93	2.79
	4	High-calorie and high-carbohydrate intake	0.19	4.13	3.71
	7	Eating too many sweets and chocolates	0.04	4.07	3.57
	8	Fizzy drinks and high-calorie energy drinks	0.09	4.13	3.77
	9	Lack of awareness of healthy eating	0.25	3.8	4
	10	Insufficient water consumption	0.13	3.86	4
	11	Badly organised mealtimes	0.05	3.67	3.62
	12	Psychological issues lead to overeating	0.06	3.33	3.36
	13	Addiction to electronic devices	0.11	3.73	3.36
	15	Sedentary lifestyles associated with electronic devices	0.23	4.07	3.67
	18	Lack of sleep	0.08	3.73	3.67
	21	Increasing portion sizes of meals	0.18	3.87	3.67
**4- School environments**			**0.22**	**4.11**	**3.89**
	14	Awareness of importance of physical activity	0.22	4	3.85
	19	Fast-food restaurants	0.14	4.07	3.93
	27	Drink water at school	0.13	4	4
	29	Insufficient physical activity	0.31	4.33	4
	34	Promote family awareness	0.17	4.29	3.93
	35	Check-ups for general health	0.36	4	3.6
**5-Home environment**			**0.03**	**3.94**	**3.90**
	20	Eat a healthy diet	0.03	4	4
	22	Consumption of sugar	0.06	3.79	3.87
	23	Consumption of additives and preservatives	0.03	3.69	3.67
	24	Eat fruit and vegetables	0	4.27	4.14
	25	Eat regular meals	0	3.79	3.93

26	Eat home-cooked meals	0.03	3.71	3.73
28	Follow Islamic rules in diet habits	0.03	4.2	3.93
30	Snacks in front of the TV	0	3.93	3.93
31	Time spent watching TV	0	3.93	3.87
33	Awareness of sleep as a healthy behaviour	0.07	4.07	4

Clusters on feasible priorities for the content of a school-based programme

**1- Ministry of Education support**		**0.07**	**4.03**	**3.98**
1	Offer healthy food in the canteen	0.08	4.67	4.13
2	Remove chocolate and sweets	0.02	4.6	4.27
7	Have a dietitian to advice the canteen	0.15	4.4	4.2
8	Establish connection between health centre and the canteen	0.03	4.47	4.27
11	Offer physical activity classes in the school	0.03	3.93	3.86
13	Attractive healthy meals in school	0.04	4.33	4
23	Educate children through the curriculum	0.06	3.6	4
24	Cooperation between the Ministry of Education and health centres	0.03	3.67	4.07
28	Canteen staff responsibility	0.01	3.93	4.07
29	Nutritionists should be part of the programme	0.04	4.07	4.2
31	Lack of physical activity facilities in the school	0.06	3.67	3.67
32	Lack of healthy and suitable diets	0.05	4.14	4.07
33	Lack of specialist school meal supervisors	0	4	3.93
34	Start physical activity classes	0.03	3.67	3.86
35	Lack of space and equipment for physical activity	0.02	3.8	3.73
40	Lack of support for such programmes	0.33	4	3.87
41	Lack of links with specialist companies to provide healthy options.	0.15	3.73	3.8
42	Prepare schools to run this programme	0.12	3.87	3.67
**2- Public health programmes**		**0.46**	**3.79**	**3.84**
3	Awareness of unhealthy foods such as fast foods	0.58	4.36	4.29
16	Courses on Mental health and healthy body	0.44	3.36	3.67
20	Workshops on combating childhood obesity	0.37	3.67	3.6
**3 -Schools Environment**		**0.14**	**3.83**	**3.92**
4	Educate children about healthy foods	0.11	4	4
5	Educate children about obesity	0.11	4.47	4
6	Offer practice programme	0.2	4.27	4.07

17	Obesity awareness through family meeting in school	0.08	3.53	3.8
18	Use brochures and films to promote awareness	0.22	3.47	3.29
21	Competitions for healthiest breakfast	0.08	3.53	3.87
22	Competitions between classes for healthiest diets	0.08	3.47	3.87
25	Set up a committee of school health administrators	0.07	3.87	4.27
26	Health guides in schools	0.21	4.07	4.2
27	Designated teachers and student advisors	0.12	3.93	4.07
36	Address the time limitations in the school curriculum	0.26	3.53	3.67
39	Extra burden on the teaching staff	0.13	3.86	3.93
**4- Wider environmental influences**		**0.7**	**3.96**	**3.82**
9	Educate children understand food labels	0.47	3.73	3.14
10	Nutrition festivals	0.51	3.47	3.33
12	Encourage healthy breakfasts	0.85	4.47	4.13
14	Encourage walking	0.63	4.4	4.13
15	Encourage healthy sleeping	0.74	3.87	4.07
19	Use social media to increase the awareness	0.69	3.67	3.73
30	Promote family responsibility	0.55	4	3.93
37	Lack of knowledge in society about obesity	0.87	3.93	3.93
38	Lack of family knowledge about obesity	1	4.07	3.93

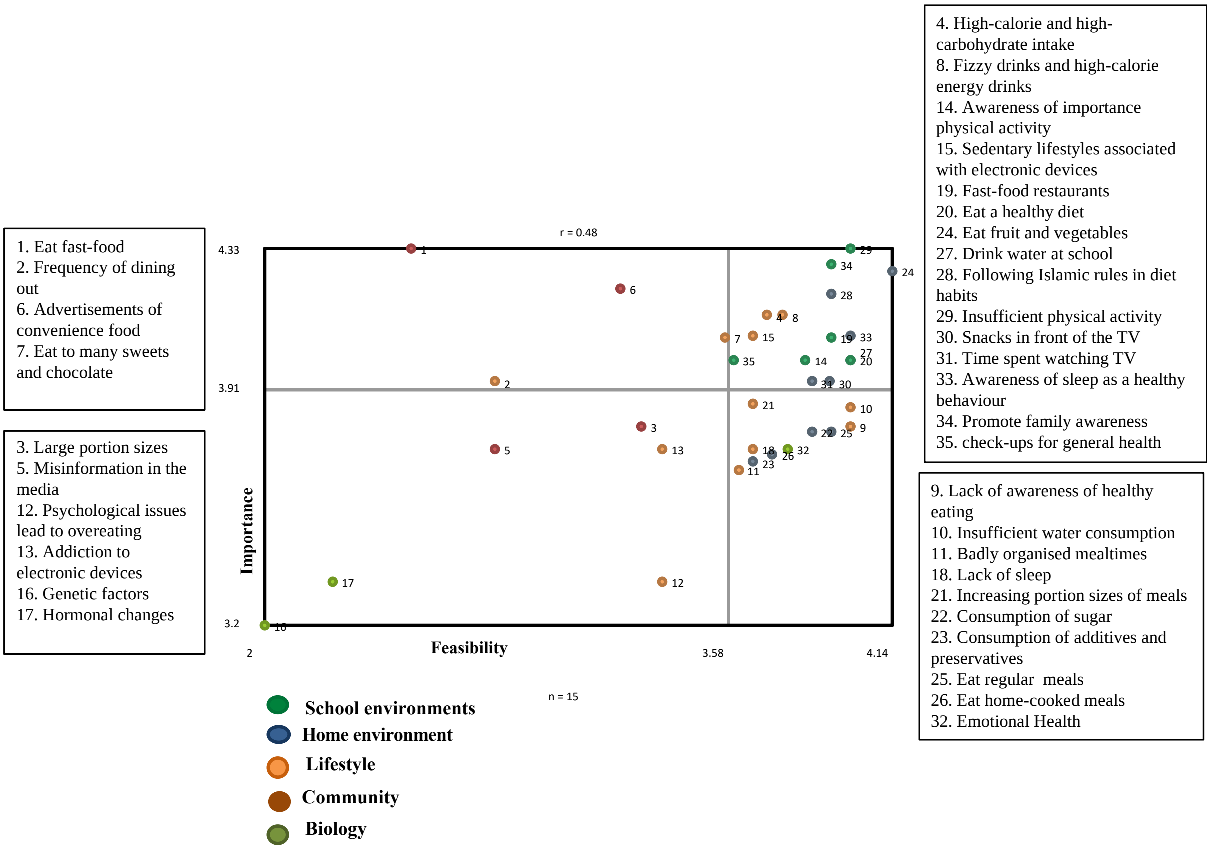

Online Figure 2. Go-zone map based on adults' perspectives on the factors that influence adolescent obesity
